# The allergic phenotype during the first 10 years of life in a prospective cohort

**DOI:** 10.1002/iid3.255

**Published:** 2019-06-17

**Authors:** Sophia Björkander, Jenny Hallberg, Jan‐Olov Persson, Gunnar Lilja, Caroline Nilsson, Eva Sverremark‐Ekström

**Affiliations:** ^1^ Department of Molecular Biosciences, The Wenner‐Gren Institute Stockholm University Stockholm Sweden; ^2^ Department of Clinical Science and Education Södersjukhuset, Karolinska Institutet Stockholm Sweden; ^3^ Sachs’ Children and Youth Hospital Stockholm Sweden; ^4^ Institute for Environmental Medicine, Karolinska Institutet Stockholm Sweden; ^5^ Department of Mathematics Stockholm University Stockholm Sweden

**Keywords:** allergy, birth season, childhood, early predictors of allergy, FeNO, lung function, parental allergy, prospective cohort

## Abstract

**Background:**

Heredity and environmental parameters jointly affect allergy development. Here, we used a Swedish prospective cohort to study the influence of heredity and factors usually associated with allergic disease and the development of allergic manifestations in combination with immunoglobulin E (IgE) sensitization at four different time points until 10 years of age.

**Methods:**

Parents‐to‐be were characterized concerning allergy and their children (n = 281) were divided based on allergic heredity and followed from birth and clinically examined for IgE‐associated allergic symptoms until 10 years of age. The relation between allergy and early‐life parameters was analyzed by logistic regression. Group‐wise comparisons were made by nonparametrical tests.

**Results:**

Early life eczema and/or asthma in combination with IgE sensitization, was a strong indicator of allergy at a later time point. Further, the early occurrence of multiple allergic symptoms among IgE‐sensitized children predisposed for a more complex allergic phenotype at later ages, independently of allergic heredity. At 10 years of age, allergic children had higher fractional exhaled nitrogen oxide (FeNO) levels, regardless of asthma, and FeNO levels were also influenced by heredity. Birth season was strongly associated with allergy development, but only in children with two allergic parents.

**Conclusion:**

Allergic eczema/asthma in early life, being born during the autumn/winter, having multiple allergic symptoms and two allergic parents were all strong predictors for having allergic diseases at 5 and 10 years of age. However, the allergic march seems to be independent of heredity, as IgE‐mediated allergies follow the same trajectories in children with and without allergic heredity.

## 1 INTRODUCTION

In Sweden, it is estimated that around 30% of the population suffer from allergy and the proportion of children suffering from symptoms varies between 5% and 20% depending on the allergic disease, according to the Swedish Asthma and Allergy Association. The increased risk of IgE sensitization and allergy development in children with allergic parents and/or siblings suggest a strong genetic contribution^1^, ^2^ including genes involved in sensing our environment and different immune cell trajectories.^3^ In addition, several environmental exposures—particularly in early life but also prenatally—have been implicated in IgE sensitization and allergy development. Factors include those related to our microbiome such as delivery mode, antibiotic usage, feeding regimes and farm environment, but also allergen exposures, and exposure to tobacco smoke, air pollution as well as dietary factors and so forth^4^ Also, the season of birth has been suggested to be of importance for an allergy‐prone immune profile, but whether it is a proxy for exposures during fetal or neonatal life is not clear.^5^, ^6^

The immune system undergoes significant maturation during childhood and it is evident from several epidemiological studies that the first 10 years of life are special in terms of the IgE profile and clinical manifestations; hence the term “allergic march” is often used to describe this phenomenon.^7^, ^8^ The mechanisms of the allergic march are still poorly understood and longitudinal investigations of clinical allergy in cohorts where both IgE sensitization and doctor's diagnosed allergy are considered are most important for understanding. Many studies to date, are cross‐sectional and survey‐based regarding allergic symptoms. Also, as most epidemiological studies primarily include children with a high allergy risk, we still know very little about allergy development in low‐risk children.

Here, we made a longitudinal approach and investigated allergy development up until 10 years of age in a Swedish prospective cohort where we had the possibility to investigate the influence of both heredity and environmental parameters commonly associated with IgE sensitization, allergic symptoms and allergy development in infancy.

## 2 MATERIALS AND METHODS

### 2.1 The prospective cohort

This study investigated the subjects from a prospective cohort of 281 children born into the cohort between 1997 and 2000 in Stockholm, Sweden. The cohort has been described in detail elsewhere.^9^, ^10^, ^11^ Briefly, families living in Stockholm and expecting a child were invited to participate in the study. The invitation was addressed to families where the mother and the father were allergic, where only the mother but not the father had allergy or where none of the parents had allergies. The allergic status of the parents‐to‐be was characterized and only those who had skin prick test (SPT) results that confirmed a negative or positive medical history of allergy to pollen/furred pets were included in the study. All infants were born at hospitals in the Stockholm area, were full‐term and had birth weights within the normal range (data not shown). The 281 children were followed from birth and were clinically examined and sampled at 1, 2, 5, and 10 years of age by the one and same paediatrician (CN).

### 2.2 Ethical statement

The study was approved by the Human Ethics Committee at Huddinge University Hospital, Stockholm (Dnr 75/97, 113/97, 331/02, 2007/858‐31/2) and the parents provided informed verbal consent. No written documentation of the participants informed approval was required, which was agreed to by the Human Ethics Committee and was according to the regulations at the time of the initiation of the study.

### 2.3 Immunoglobulin E sensitization and allergy diagnosis

At the age of 1, 2, 5, and 10 years of age, SPT was performed against food (egg white, cod, peanut, cow's milk, and soybean) and inhalant (cat, dog, *Dermatophagoides farinae*, birch, and timothy) allergens according to the manufacturer's instructions (ALK, Copenhagen, Denmark). Histamine chloride and allergen diluent serving as positive and negative controls, respectively. Mugworth, horse, rabbit, and hazelnut were included only at 10 years of age. The SPT was considered positive if the wheal diameter was greater than or equal to 3 mm after 15 minutes. Serological analysis of allergen‐specific IgE‐antibody (sIgE‐ab) to the selected allergens was performed using ImmunoCAP (Phadia AB; [Thermofisher], Uppsala, Sweden) and levels greater than or equal to 0.35 kU/l were classified as positive. Children were considered to be sensitized if positive for at least one SPT and/or sIgE‐ab and were classified as allergic if a positive SPT or/and sIgE‐ab was accompanied by one or several allergic symptom(s) (eczema, food allergy, asthma, and rhinoconjunctivitis). Sensitized children without allergic symptoms as well as nonsensitized children with symptoms were considered as nonallergic. One child at 1 year of age and two children at 10 years of age were excluded from the analysis due to uncertain allergy diagnosis. At each visit, the presence of allergy‐related diseases was recorded for each child. The definitions for allergic diseases were: *Eczema* was defined according to Hanifin and Rajka.^12^ *Asthma* was defined according to the Swedish Pediatric Allergy Society guidelines. *Asthma up to the age of 2 years* was defined as three or more episodes of wheezing or signs of hyperreactivity, or any episode of wheezing or hyperreactivity if combined with a family history of allergic disease or allergic symptoms in the child or respiratory symptoms treated with inhaled steroids. After 2 years of age, any episode of wheezing or signs of hyperreactivity reaction or wheezing after exposure to an allergen or respiratory symptoms treated with inhaled steroids was defined as *asthma*. *Food allergy* was diagnosed as acute onset of symptoms such as skin reactions, wheezing, vomiting or diarrhea, on more than one occasion after ingestion of, or contact with, a particular food allergen. *Rhinoconjunctivitis* was defined as symptoms of rhinitis and/or conjunctivitis appearing at least twice after exposure to a particular allergen and unrelated to infection.

### 2.4 Measurements of lung function and fractional exhaled nitrogen oxide

The lung function was evaluated by spirometry at the age of 10 years, using the Jaeger MasterScreen‐IOS system (Carefusion Technologies, San Diego, CA). All subjects performed several maximum expiratory flow‐volume (MEFV) measurements from which the highest values of forced vital capacity (FVC), forced expiratory volume in 1 second (FEV1) were extracted and used from analysis, provided that MEFV curve passed quality inspection and that the two highest FEV1 and FVC readings were reproducible according to American Thoracic Society/European Respiratory Society (ATS/ERS) criteria.^13^ Values were converted into *z*‐scores using the GLI‐reference equations.^14^ Fractional exhaled nitrogen oxide (FeNO) was measured at 10 years of age according to ATS/ERS criteria,^15^ using the NIOX MINO (Aerocrine AB, Solna, Sweden).

### 2.5 Statistical analysis

The relations between allergy and early‐life parameters were analyzed with logistic regression in both cross‐sectional and longitudinal ways, adjusting for parental allergy, and are presented in the tables. The *χ* ^2^ test followed by Fisher's exact test, or the Mann‐Whitney *U* test were used for nonparametrical group‐wise comparisons as presented in all figures and Table 2. The GraphPad Prism 6 software (GraphPad Software, San Diego, CA) was used for data presentation. Scatter dot plots show medians with range. Results were considered significant if *P* < 0.05.

## 3 RESULTS

### 3.1 Longitudinal assessment of IgE‐mediated allergy in relation to heredity, and early‐life parameters usually associated with allergic disease

**Figure 1:**
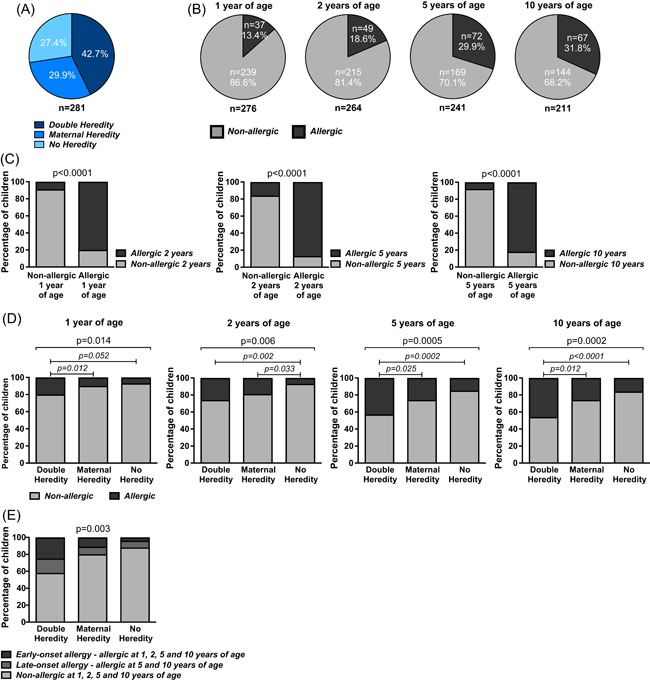
Longitudinal assessment of IgE‐mediated allergy in relation to heredity in a Swedish prospective birth cohort. A, The cohort consists of 281 children divided into three heredity groups based on confirmed parental allergy. B, The percentages of children with confirmed IgE‐mediated allergic disease at 1, 2, 5, or 10 years of age. C, The percentages of nonallergic and allergic children at 2, 5, or 10 years of age in relation to having been allergic or nonallergic at 1, 2, or 5 years of age respectively. D, The percentage of children with IgE‐mediated allergic disease within each heredity group at 1, 2, 5, or 10 years of age. E, The percentages of children with early‐ and late‐onset allergy in relation to heredity. The *χ* ^2^ test followed by Fisher's exact test were used for statistical analyses

The 281 children were followed prospectively from birth until 10 years of life and were divided into three groups based on parental allergy: “No Heredity” group (NH, n = 77), “Maternal Heredity” group (MH, n = 84), and “Double Heredity” group (DH, n = 120) (Figure 1A). We first assessed allergy in general terms, defined as at least one allergic symptom together with a positive SPT and/or circulating sIgE‐ab. In the total cohort, allergy increased from 13.4% at 1 year of age to 31.8% at 10 years of age (Figure 1B and Table 1A). The follow‐up frequency was very high and equal among the heredity groups (Table 1B). Being allergic at 1, 2, or 5 years of age was strongly associated with remaining allergies at 2, 5, and 10 years of age, respectively (Figure 1C).

**Table 1:** 

A. *Allergy prevalence*				
	1 year of age	2 years of age	5 years of age	10 years of age
Total cohort	37/276 (13.4%)	49/264 (18.6%)	72/241 (29.9%)	67/211 (31.8%)
No Heredity	5/75 (6.7%)	5/72 (6.9%)	10/66 (15.2%)	9/58 (15.6%)
Maternal Heredity	8/82 (9.6%)	15/79 (19.0%)	19/74 (25.7%)	16/62 (25.8%)
Double Heredity	24/119 (20.2%)	29/113 (25.7%)	43/101 (42.6%)	42/91 (46.2%)

B. *Follow‐up frequency*				
	1 year of age	2 years of age	5 years of age	10 years of age
Total cohort	276/281 (98.2%)	264/281 (94.0%)	241/281 (85.8%)	211/281 (75.1%)
No Heredity	75/77 (97.4%)	72/77 (93.5%)	66/77 (85.6%)	58/77 (71.4%)
Maternal Heredity	82/84 (97.6%)	79/84 (94.0%)	74/84 (88.1%)	62/84 (73.8%)
Double Heredity	119/120 (99.2%)	113/120 (94.2%)	101/120 (84.2%)	91/120 (75.8%)

As expected, parental allergy was a strong risk factor for allergic disease (Figure 1D). Two allergic parents was associated with the highest risk and further associated with an early allergy onset and persistence (Figure 1E).

We further evaluated other factors that have been reported to relate to allergic disease in childhood (Table 2). In relation to parental allergy, there were no differences regarding sex, delivery mode, the presence of siblings at birth, exclusive (0–3 or >3 months) or partial (0–8 or >8 months) breast feeding, while there was a significantly lower proportion of pet owners in the DH group (Table 2A). When further evaluating the same parameters in relation to IgE‐mediated allergy in the children, using a logistic regression model adjusted for heredity, we did not find any significant associations between these parameters and allergy (Table 2B). A longitudinal analysis did not alter these findings (data not shown).

**Table 2:** 

A. *Demographic data*						
	Sex (female/male)	Delivery mode (cesarean/vaginal)	Siblings at birth (yes/no/missing)	Pets at birth (yes/no)*	Exclusive breast feeding (0‐3 mo/ > 3 mo/missing)	Partial breast feeding (0‐8 mo/ > 8 mo/missing)
Total cohort (n = 281)	140/141	42/239	111/135/35	44/237	48/232/1	111/145/25
No Heredity (n = 77)	37/40	8/69	34/35/8	20/57* ^,^ **	12/64/1	29/44/4
Maternal Heredity (n = 84)	40/44	16/68	35/40/9	15/69* ^,^ ***	16/68/0	34/40/10
Double Heredity (n = 120)	63/57	18/102	42/60/18	9/111* ^,^ ** ^,^ ***	20/100/0	48/61/11

*B. Early‐life parameters and allergy prevalence*					
	Allergic/total, %	Allergic/total, %	Fisher's exact test	OR (*P*)	OR_adj_ ****(*P*)
*Sex*	Girl	Boy			
1 year of age	18/137 (13.1)	19/139 (13.7)	ns	0.96 (0.897)	0.90 (0.762)
2 years of age	20/129 (15.1)	29/135 (21.5)	ns	0.67 (0.213)	0.63 (0.158)
5 years of age	37/116 (31.9)	35/125 (28.0)	ns	1.20 (0.509)	1.16 (0.609)
10 years of age	38/110 (34.5)	29/101 (28.9)	ns	1.31 (0.364)	1.27 (0.436)
*Delivery mode*	Vaginal	Caesarean			
1 year of age	31/233 (13.3)	6/43 (14.0)	ns	1.09 (0.856)	1.07 (0.891)
2 years of age	45/223 (20.2)	4/41 (9.8)	ns	0.44 (0.140)	0.41 (0.109)
5 years of age	60/202 (29.7)	12/39 (30.8)	ns	1.10 (0.803)	1.09 (0.827)
10 years of age	55/181 (30.4)	12/30 (40.0)	ns	1.63 (0.234)	1.54 (0.315)
*Siblings at birth*	Yes	No			
1 year of age	16/110 (14.5)	17/135 (12.6)	ns	1.18 (0.656)	1.29 (0.512)
2 years of age	18/111 (16.2)	29/135 (21.5)	ns	0.71 (0.297)	0.75 (0.389)
5 years of age	30/104 (28.9)	41/122 (33.6)	ns	0.80 (0.443)	0.89 (0.686)
10 years of age	27/91 (29.7)	37/105 (35.2)	ns	0.78 (0.408)	0.87 (0.654)
*Pets at birth*	Yes	No			
1 year of age	2/41 (4.5)	35/235 (14.9)	ns	0.29 (0.101)	0.38 (0.200)
2 years of age	4/39 (10.3)	42/225 (18.7)	ns	0.46 (0.157)	0.59 (0.351)
5 years of age	8/37 (21.6)	64/204 (31.4)	ns	0.60 (0.237)	0.81 (0.646
10 years of age	7/31 (22.6)	60/180 (33.3)	ns	0.58 (0.239)	0.82 (0.680)
*Exclusive breast feeding*	0‐3 mo	>3 mo			
1 year of age	8/47 (17.0)	29/229 (12.7)	ns	0.71 (0.426)	0.70 (0.418)
2 years of age	8/44 (18.2)	41/220 (18.6)	ns	1.03 (0.944)	1.02 (0.962)
5 years of age	15/38 (39.5)	57/203 (28.1)	ns	0.60 (0.162)	0.50 (0.071)
10 years of age	13/33 (39.4)	54/178 (30.3)	ns	0.67 (0.307)	0.57 (0.174)
*Partial breast feeding*	0‐8 mo	>8 mo			
1 year of age	17/111 (15.3)	17/144 (11.8)	ns	0.85 (0.575)	0.84 (0.542)
2 years of age	21/106 (19.8)	24/140 (17.1)	ns	0.95 (0.860)	0.94 (0.801)
5 years of age	34/98 (34.7)	32/126 (25.4)	ns	0.81 (0.376)	0.76 (0.262)
10 years of age	31/89 (34.8)	29/106 (27.4)	ns	0.95 (0.844)	0.91 (0.704)

*Note*: Missing: information is not available.

Abbreviation: ns, non significant.

* *P*   =  0.002 (*χ* ^2^ test).

** *P* < 0.001 (Fisher's exact test).

*** *P* = 0.028 (Fisher's exact test).

**** OR_adj_: Adjusted for parental allergy

### 3.2 Season of birth is strongly associated with being allergic, particularly among children in the DH group

**Figure 2:**
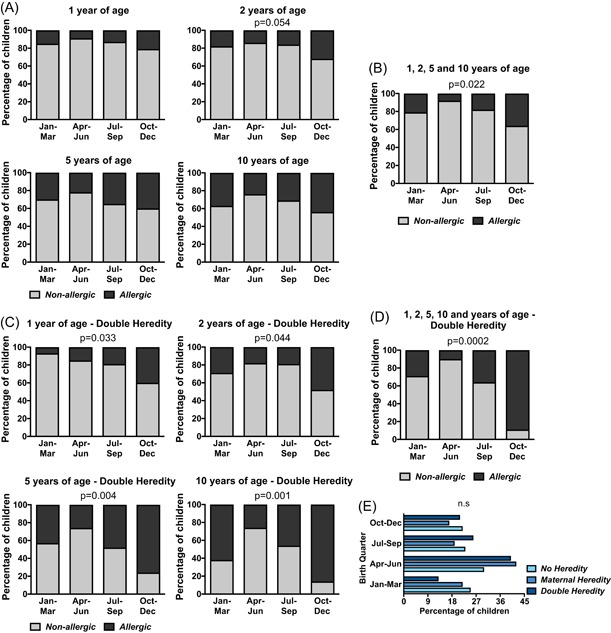
Season of birth and its association with IgE‐mediated allergy. A and B, The percentages of allergic children at 1, 2, 5, or 10 years of age in relation to birth season. C and D, The percentages of allergic children at 1, 2, 5, or 10 years of age in relation to birth season among the children with two allergic parents (Double Heredity, DH). E, Birth season in relation to allergic heredity as such. The *χ* ^2^ test was used for statistical analyses

We also investigated whether the season of birth related to allergy in the cohort. A birth period between October and March seemed to be overrepresented among allergic children in general (Figure 2A and 2B). When we further analyzed this in relation to allergic heredity, we found a strong association between a birth period between October and December and future IgE‐mediated allergy among the children in the DH group (Figure 2C and 2D). This observation was also confirmed by the longitudinal logistic regression model (*P* = 0.043). Importantly, there were no differences in birth season between the three heredity groups as such (Figure 2E).

### 3.3 The allergic march is independent of allergic heredity

**Figure 3:**
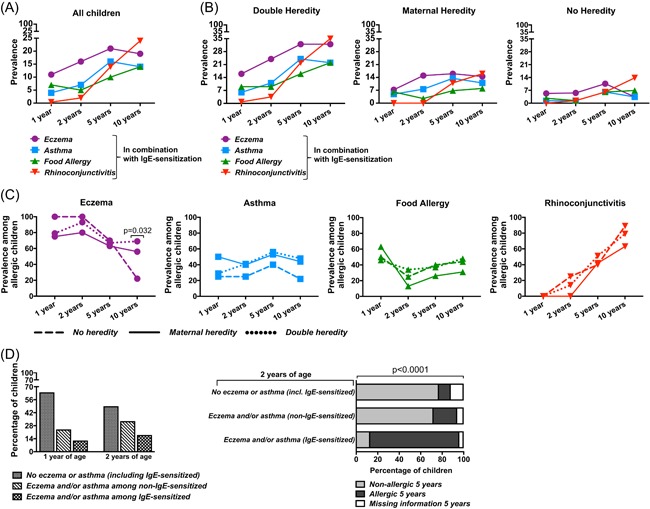
The allergic march in relation to parental allergy. A, The prevalence of eczema (purple), asthma (blue), food allergy (green), and rhinoconjunctivitis (red) in combination with IgE sensitization among all children at 1, 2, 5, or 10 years of age. B, The prevalence of eczema, asthma, food allergy, and rhinoconjunctivitis in combination with IgE sensitization among all children at 1, 2, 5, or 10 years of age within each heredity group. C, Comparison of the prevalence of eczema, asthma, food allergy, and rhinoconjunctivitis over time among the IgE‐sensitized children within the three different heredity groups. D, Left: The frequency of IgE sensitization among children with eczema and/or asthma at 1 or 2 years of age. The proportion of children without or with eczema and/or asthma in relation to IgE sensitization. Right: Eczema and/or asthma with or without IgE sensitizations at 2 years of age as early predictors of persistent allergy. The *χ* ^2^ test was used for statistical analyses

When examining the different allergic symptoms among all the children in the cohort, allergic eczema was the most common symptom at 1, 2, and 5 years of age when the prevalence peaked at 21%. The prevalence of allergic asthma peaked at 5 years of age (16%), and the prevalence of food allergy varied from 7% at 1 year of age to 14% at 10 years of age. Allergic rhinoconjunctivitis was very rare at 1 and 2 years of age, but the most common symptom at 10 years of age (24%) (Figure 3A).

As expected, the prevalence of each symptom in combination with IgE sensitization was highest in the DH group and lowest in the NH group. Still, all three heredity groups showed the same pattern with eczema being most common between 1 and 5 years of age and rhinoconjunctivitis most common at 10 years of age. In all three groups, food allergy had the lowest prevalence at 2 years of age, to then increase (Figure 3B). We further followed the patterns of the investigated symptoms in IgE‐sensitized children for each heredity group over the 10‐year period (Figure 3C). The prevalence of each symptom over time was strikingly similar within all the three heredity groups, apart from eczema at 10 years of age (Figure 3C).

Finally, we investigated whether eczema and asthma were predictors for future allergy. Notably, it was more common to have eczema and/or asthma in the absence of IgE sensitization at 1 and 2 years of age than being IgE sensitized and having symptoms (Figure 3D, left). Still, it was very clear that it was early life allergic eczema and/or asthma (ie*,* being IgE‐sensitized and having symptoms) that was a strong predictor of allergy at a later time point—here shown for 5 years of age, while these symptoms without a simultaneous IgE sensitization was not (Figure 3D, right).

### 3.4 Early occurrence of multiple allergic symptoms predisposes for a more complex allergic phenotype at later ages, but does not relate to allergic heredity

**Figure 4:**
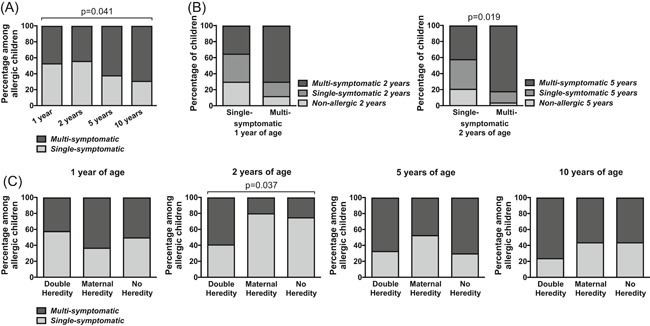
Multiple allergic symptoms at early ages among IgE‐sensitized children and later allergy. A, The proportion of allergic subjects with single or multiple symptoms at each time point. B, The proportion of children that were nonallergic, single‐symptomatic or multisymptomatic at 2 or 5 years of age in relation to having been single or multisymptomatic at 1 or 2 years of age, respectively. C, The proportion of allergic subjects with single or multiple symptoms within each heredity group at each time point. The *χ* ^2^ test was used for statistical analyses

To further characterize the complexity of allergic diseases, we investigated whether allergic children had one (single‐symptomatic) or several allergic symptoms (multisymptomatic). The percentage of multisymptomatic subjects was 47.2% at 1 year of age and increased to 68.7% at 10 years of age (Figure 4A and Table 3). The most common symptom combinations among the multisymptomatic IgE‐sensitized children are summarized in Table 3.

**Table 3:** Prevalence of symptoms among IgE‐sensitized children

1 year of age (n = 36)	%	2 years of age (n = 48)	%	5 years of age (n = 72)	%	10 years of age (n = 67)	%
**Single‐symptomatic**	**52.8**	**Single‐symptomatic**	**56.2**	**Single‐symptomatic**	**37.5**	**Single‐symptomatic**	**31.3**
Eczema	68.4	Eczema	81.5	Eczema	55.6	Eczema	57.1
Asthma	15.8	Asthma	14.8	Asthma	22.2	Asthma	28.6
Food allergy	15.8	Food allergy	3.7	Food allergy	14.8	Food allergy	9.5
Rhinoconjunctivitis	0.0	Rhinoconjunctivitis	0.0	Rhinoconjunctivitis	7.4	Rhinoconjunctivitis	4.8
**Multisymptomatic**	**47.2**	**Multisymptomatic**	**43.8**	**Multisymptomatic**	**62.5**	**Multisymptomatic**	**68.7**
Eczema‐food‐rhino	41.2	Asthma‐eczema	28.6	Asthma‐eczema	20.0	Eczema‐food‐rhino	19.6
Eczema‐food	41.2	Asthma‐eczema‐food	28.6	Asthma‐eczema‐food‐rhino	17.8	Asthma‐eczema‐food‐rhino	19.6
Eczema‐rhino	5.9	Eczema‐food	19.1	Eczema‐rhino	13.3	Eczema‐rhino	17.4
Asthma‐food	5.9	Eczema‐rhino	9.5	Asthma‐eczema‐rhino	13.3	Asthma‐eczema‐rhino	10.9
Asthma‐eczema	5.8	Asthma‐eczema‐food‐rhino	9.5	Eczema‐food‐rhino	8.9	Asthma‐food	8.7
		Asthma‐eczema‐rhino	4.7	Asthma‐rhino	6.7	Asthma‐food‐rhino	8.7
				Asthma‐food‐rhino	6.7	Asthma‐rhino	6.5
				Asthma‐eczema‐food	4.4	Asthma‐eczema	4.4
				Food‐rhino	4.4	Food‐rhino	2.1
				Asthma‐food	2.3	Eczema‐food	2.1
				Eczema‐food	2.2		

Being multisymptomatic at 1 year of age clearly associated with remaining multisymptomatic at 2 years of age, and the same pattern was observed between 2 and 5 years of age (Figure 4B). In addition, there was a higher proportion of children that outgrew their IgE‐mediated allergy within the single‐symptomatic groups (Figure 4B). Notably, allergic heredity was not linked to being multisymptomatic, except at 2 years of age (Figure 4C).

### 3.5 FeNO levels but not lung function at 10 years of age relate to allergy and heredity

**Figure 5:**
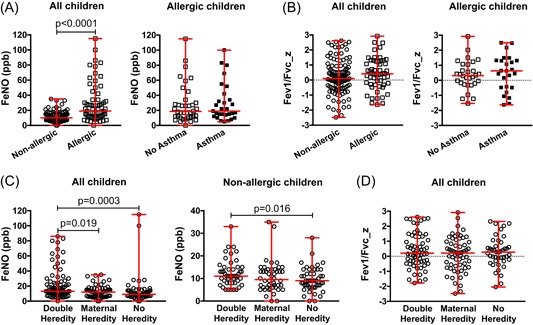
FeNO and lung function at 10 years of age. A, Exhaled FeNO levels at 10 years of age in relation to allergy among all children (left) and in relation to asthma among allergic children (right). B, Lung function, measured as FEV1/FVC *z*‐scores, in relation to allergy among all children (left) and in relation to asthma among allergic children (right). C, Exhaled FeNO‐levels at 10 years of age among all children (left) and among nonallergic children (right) within each heredity group. D, Lung function, measured as FEV1/FVC *z*‐scores, at 10 years of age for all children within each heredity group. The Mann‐Whitney *U*‐test was used for statistical analyses. FeNO, fractional exhaled nitric oxide; FEV1, forced expiratory volume/second; FVC, forced vital capacity

At 10 years of age, the median level of FeNO was higher among allergic than nonallergic children (Figure 5A, left), but did not differ between allergic children with and without asthma (Figure 5A, right). However, there was no difference in FEV1/FVC *z*‐scores between the allergic and nonallergic group (Figure 5B, left), or between children with or without allergic asthma (Figure 5B, right). Allergic heredity associated with the FeNO levels, and interestingly this was visible also when only including nonallergic children (Figure 5C), while the FEV1/FVC *z*‐scores was similar between all heredity groups (Figure 5D).

## 4 DISCUSSION

In the present study, we followed IgE‐mediated allergy development, in children born into the cohort, during the first 10 years of life. The parents‐to‐be were carefully investigated concerning allergy and we monitored allergic symptoms and IgE‐sensitization in their children in relation to heredity and different early‐life parameters.

In our cohort, the prevalence of having any allergic disease increased with age and corresponded to numbers presented in a large Swedish population‐based cohort.^1^ We observed that the prevalence of food allergy dropped between 1 and 2 years of age, to then increase up to 10 years of age, without any tendency to level off. We suggest that the initial drop is due to that children outgrow their milk and/or egg allergy, but later develop allergies to other foods, such as peanut.^16^ Early allergic eczema has been demonstrated to be a good predictor of future allergic disease,^17^, ^18^ a finding that agrees well with the atopic march concept. Although the prevalence of allergic eczema was high at 1 year of age, allergic asthma and food allergy was also relatively prevalent and around half of the allergic children had more than one symptom. Hence, we are not able to elucidate the predictive value of early allergic eczema alone on future allergy in our cohort. However, we observed that early allergic eczema and/or asthma at the age of 2 years was a strong predictor of allergic disease at later ages, in contrast to early eczema/asthma in the absence of IgE‐sensitization. It could, therefore, be of importance to investigate IgE‐sensitization in children with eczema at early ages to estimate future allergy diseases.

When the present cohort was initiated, it was generally believed that maternal allergy posed a bigger risk for the offspring to become IgE‐sensitized and allergic.^19^, ^20^ Therefore, our study cohort included three heredity groups; double (DH), maternal (MH), or no allergic heredity (NH). DH associated the highest degree of allergy development in the offspring, in agreement with the results from several other studies.^21^, ^22^ Maternal allergy posed an intermediate risk, stressing that paternal allergy should not be neglected.^23^, ^24^ We observed that being allergic at an early age was strongly associated with being allergic later in life, in line with a previous study where the only significant predictors of allergic sensitization were a family history of allergic disease and earlier allergic symptoms.^25^

Comorbidities are common in allergic individuals, as the cooccurrence of allergic asthma, rhinitis, and eczema are more common than expected by chance.^26^ Indeed, having more than one allergic symptom early in life associated with remaining multisymptomatic also in our study. Importantly, we observed that heredity did not associate with being multisymptomatic or with a higher prevalence or particular symptoms. This suggests that the course and severity of established allergy follow the same trajectories in children with and without allergic heredity.

The surrounding milieu in early‐life is frequently pointed out as a key risk factor as well as a driver of allergy.^27^ In our cohort, we could not find any association between sex, siblings, breastfeeding, pets, and mode of delivery with any investigated aspect of allergy development, in agreement with several other studies.^28^, ^29^, ^30^, ^31^ Indeed, the influence of these parameters on allergy could be highly variable between cohorts due to factors difficult to control for, for example, for pets: indoor/outdoor; type of pet/breed and so forth. Here cohort size will also influence, and the number of children in our cohort were insufficient to evaluate this in depth. However, we report a clear significant association between birth season and allergic symptoms in the DH group; being born in October‐December was overrepresented. This is interesting to note, as there was no difference in birth season between the three heredity groups as such. We do not have a clear explanation for this finding, but we could speculate that an increased prevalence of respiratory infections during the children's first months of life, and more time spent indoors during this dark and cold period in Sweden, could have contributed. However, previously published reports on birth season and allergy show different results.^5^, ^6^, ^32^, ^33^ This might be due to geographical variations, as well as the allergic subtype studied. For example, the risk of eczema has been shown to be higher in children born in the autumn, and this was associated with the DNA methylation pattern in whole blood, suggesting that seasonal exposures might trigger epigenetic changes of immune reactivity.^34^ Cord blood immune responsiveness seems to vary with birth season, but whether these differences persist during childhood is not known.^35^

FeNO is considered as a marker of T helper 2‐associated inflammation, but its benefits as a noninvasive biomarker for asthma management are not entirely clear.^36^, ^37^ An interesting observation in our study was that allergic 10‐year‐olds had higher FeNO levels than those who were nonallergic regardless of asthma, also supported by others.^38^ Allergic heredity seems to be important for FeNO levels illustrated by the fact that the DH group had higher FeNO levels regardless of being allergic or nonallergic. This is in line with a twin study which showed that variations in FeNO appear to be explained by different genetic influences.^39^ Altogether, the data suggests that increased levels of FeNO seem to be a marker for a general allergic inflammation and a genetic predisposition for allergy. We did not see an effect of neither asthma nor allergy on lung function measured by spirometry. This is not too surprising as our cohort includes very few individuals with severe asthma and the fact that allergy as such does not influence lung function.^40^

Our cohort, where the children were born into the cohort by parents carefully characterized concerning allergic status is not a general population‐based cohort. Yet, the well‐known parental allergic status and subsequently children with different allergy risks, limits the likelihood of introducing bias and enabled us to study allergy also in low‐risk children. Also, the high follow‐up frequency and no significant differences in the follow‐up between the heredity groups at any time point gives us a robust cohort.

In conclusion, we show that strong predictors for having allergic diseases at 5‐to‐10 years are allergic eczema/asthma in early life, being born during the autumn/winter, having multiple allergic symptoms before 2 years of age and two allergic parents. However, the allergic march seems to be independent of heredity, as IgE‐mediated allergies follow the same trajectories in children with and without allergic heredity.

## CONFLICT OF INTERESTS

The authors declare that there are no conflict of interests.

## DATA ACCESSIBILITY

The data that support the findings of this study are available from the corresponding author upon reasonable request.
